# 20-Year follow-up and comparison of valved conduits used for right ventricular outflow tract reconstruction: single-centre, propensity score match analysis

**DOI:** 10.1093/icvts/ivad182

**Published:** 2023-11-09

**Authors:** Fadi Sabateen, Vladimír Soják, Aref Saif Nagi, Pavel Valentík, Michal Šagát, Matej Nosál'

**Affiliations:** Department of Pediatric Cardiac Surgery, Children’s Heart Centre, National Institute of Cardiovascular Diseases, Bratislava, Slovakia; Department of Pediatric Cardiac Surgery, Children’s Heart Centre, National Institute of Cardiovascular Diseases, Bratislava, Slovakia; Department of Pediatric Cardiac Surgery, Children’s Heart Centre, National Institute of Cardiovascular Diseases, Bratislava, Slovakia; Department of Pediatric Cardiac Surgery, Children’s Heart Centre, National Institute of Cardiovascular Diseases, Bratislava, Slovakia; Department of Pediatric Cardiac Surgery, Children’s Heart Centre, National Institute of Cardiovascular Diseases, Bratislava, Slovakia; Department of Pediatric Cardiac Surgery, Children’s Heart Centre, National Institute of Cardiovascular Diseases, Bratislava, Slovakia

**Keywords:** Congenital heart defects, Homografts, Right ventricular outflow tract reconstruction, Contegra, Matrix P Plus N, Long-term results

## Abstract

**OBJECTIVES:**

Surgical repair of complex congenital heart defects with hypoplasia or atresia of the right ventricular outflow tract (RVOT) may require pulmonary valve implantation or replacement during the primary repair or reoperation. The purpose of this study is to evaluate the outcomes of cryopreserved homografts, bovine jugular vein conduits and decellularized Matrix P Plus N conduits in patients undergoing RVOT reconstruction at a single centre.

**METHODS:**

Retrospective, single-centre review of 173 patients with 199 conduits undergoing right ventricle-to-pulmonary artery reconstruction with valved conduit from 2002 to 2022.

**RESULTS:**

A total of 199 conduits were implanted in 173 patients (62.8% male), with a mean age of 8.97 ± 8.5 years. The following 3 types of conduits were used: homografts 129 (64.8%), bovine jugular vein conduits 45 (22.7%) and Matrix P Plus N 25 (12.5%). During the mean follow-up duration of 8.6 ± 5.8 years, there were 20 deaths, 35 conduit reoperations and 44 catheter reinterventions. Overall survival, reoperation-free and catheter reintervention-free survival at 20 years were 83%, 67.8% and 65.6%, respectively. Multivariable Cox analysis identified younger patient age, smaller conduit size, low patient weight and primary diagnosis of common arterial trunk as risk factors for reoperation and catheter reintervention.

**CONCLUSIONS:**

Long-term outcomes of reconstruction of the RVOT using homografts, bovine jugular vein and Matrix P Plus N conduits were acceptable. The reoperation rate for conduit dysfunction did not differ significantly among groups. Over time, the need for conduit replacement was higher in smaller conduits and in patients with common arterial trunk diagnosis.

**Figure:**
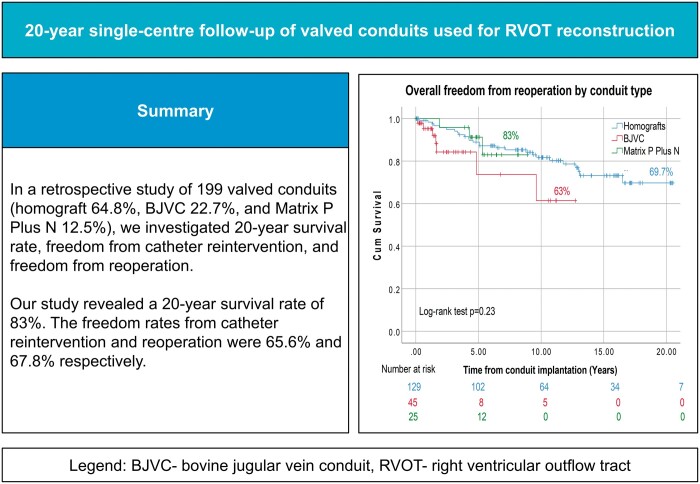

## INTRODUCTION

Right ventricle-to-pulmonary artery (RV–PA) conduits have allowed for the repair of a large number of congenital cardiac defects with hypoplasia or atresia of the right ventricular outflow tract (RVOT). These fundamental defects include common arterial trunk (CAT), transposition of the great arteries with ventricular septal defect and pulmonary stenosis or atresia, tetralogy of Fallot (TOF) with absent pulmonary valve, TOF with anomalous coronary artery crossing the RVOT, pulmonary atresia with ventricular septal defect and different forms of double-outlet right ventricular. Conduits have also made it feasible to replace the aortic root with a pulmonary autograft (Ross operation) [1].

Valved homografts, first described by Ross and Somerville [2] in 1966, have become the most commonly used valved conduit for RVOT reconstruction. The benefits of homografts (pulmonary and aortic) include ease of insertion and better hemostasis [3]. However, other studies have found that homografts are not widely available and have poor durability [4]. Furthermore, small-sized homografts are in short supply [4]. In an effort to develop more durable valved conduits for paediatric patients, alternative methods of preparation have been explored, including decellularization and the use of xenografts like the Matrix P bioprosthesis and the bovine jugular vein conduit. Bovine jugular vein conduit (BJVC), also known as Contegra, was first introduced by Medtronic Inc. (Minneapolis, MN, USA) in 1999. It contains a trileflet valve within a long conduit lumen and it is available in a variety of sizes (12–22 mm) [5, 6]. The Matrix P Plus N (AutoTissue GmbH, Berlin, Germany) is a decellularized porcine pulmonary valve, which is covered with a decellularized equine pericardial patch [7].

The goal of this study is to compare homografts, BJVC and decellularized Matrix P Plus N conduits with respect to patient survival, reoperation-free and catheter reintervention-free survival in paediatric patients and adolescents with congenital heart defects requiring primary conduit implantation or replacement for RVOT reconstruction.

## PATIENTS AND METHODS

### Ethical statement

The study was approved by the Institutional Review Board of the National Institute of Cardiovascular Diseases, Bratislava, Slovakia, on 8 February 2023 (approval number: 05/2023), and the informed consent was not required, as the study was retrospective.

### Patients

The study population consisted of patients who underwent RVOT conduit implantation or replacement at the Department of Paediatric Cardiac Surgery, Children's Heart Centre, Bratislava, Slovakia, between 1 January 2002 and 31 June 2022. The patients’ medical records were retrospectively reviewed.

Patients with congenital heart defects receiving cryopreserved homografts, BJVC (Contegra) or Matrix P Plus N conduits at the time of primary repair or reoperation were included. Exclusion criteria included patients whose medical records or operative notes were not retrievable as well as those who had an RV–PA conduit implanted and underwent heart transplantation due to myocardial dysfunction. Additionally, all other conduit types were excluded.

Demographic data, cardiac diagnosis, sex, Aristotle score [8], age at surgery for RVOT reconstruction, conduit characteristics including sizes and types, echocardiographic findings, follow-up clinical data, catheter reintervention and reoperation data were collected. We also analysed treatment outcomes, patient survival and the duration of freedom from catheter reintervention and reoperation.

The RV–PA conduits were classified into 3 groups: homograft (aortic and pulmonary), BJVC and Matrix P Plus N conduit. In Slovakia, cryopreserved homografts (aortic and pulmonary) are provided by the Central Tissue Bank at the Medical faculty of Comenius University, Bratislava. Cryopreservation was performed using Dulbecco’s Modified Eagle Medium 1% containing bovine serum and dimethylsulphoxide for preservation and homografts were stored in liquid nitrogen at −150°C.

A total of 199 conduits were implanted in 173 patients. Of these, 114 (57.2%) were pulmonary homografts, 15 (7.6%) were aortic homografts, 45 (22.7%) were BJVC and 25 (12.5%) were Matix P Plus N conduits. The median follow-up for the entire cohort was 8.4 (0.008–20.5) years. The median follow-up was 11.36 (0.008–20.5) years for homografts, 2.09 (0.18–12.7) years for BJVC and 5 (0.008–9) years for Matrix P Plus N conduits.

Propensity score-matching analysis was done, and 82 conduits (41 matched pairs, 76 patients) were chosen for comparison.

The study end points included freedom from reoperation, freedom from catheter reintervention and survival. Freedom from conduit reoperation was defined as the time period between operative conduit implantation and operative conduit replacement. Freedom from catheter reintervention was defined as the time period between operative conduit implantation and catheter intervention; balloon valvuloplasty, stent implantation or percutaneous pulmonary valve implantation (PPVI). The orthotopic placement was defined as the implantation of the conduit within the normally situated RVOT (Ross procedure), and the conduit was placed in a heterotopic position in all other patients.

Decisions about the primary operation, the reoperation or catheter reintervention were made individually in a multidisciplinary conference. Our current practice is to minimize implanting valved conduits and we prefer to use native tissue if possible in primary operations. However, we still use valved conduits in some diagnoses and surgical repairs, such as TOF with absent pulmonary valve, TOF with anomalous coronary artery crossing the RVOT, e.g. the left anterior descending artery from right coronary artery, CAT type A2–A3, CAT type A4 with interrupted aortic arch, Ross and Ross–Konno procedure, Yasui procedure and in Senning–Rastelli operation. The indications for the replacement of existing RV–PA conduits included infective endocarditis, severe conduit stenosis with a peak gradient >50 mmHg and/or severe regurgitation and conduit aneurysm or pseudoaneurysm. Echocardiography and cardiac MRI were used to evaluate the RV–PA conduit function and determine the severity of right ventricle dilatation and/or dysfunction. Bovine jugular vein (BJV) and Matrix P Plus N conduits patients received aspirin for 6 months postoperatively.

The national population register of Slovakia was used to verify the patient's survival status.

Reoperations conducted on patients following initial RV–PA conduit implantation were assessed using either our congenital heart disease database in Children’s Heart Centre, Bratislava, or external databases maintained by 3 departments of adult cardiac surgery in Slovakia. Catheter reinterventions were analysed using a registry run by the Division of Paediatric and Adult Cardiology in Bratislava, Slovakia.

### Statistical analysis

Categorical variables are presented as numbers with percentages. Comparisons for categorical variables were calculated with chi-square (χ^2^) or Fisher’s exact test. Continuous variables are expressed as means and standard deviations or medians with interquartile ranges (IQR). The Shapiro–Wilk test was used to assess the normality of the distribution. Comparisons for continuous variables in the unmatched cohort were calculated with the unpaired *t*-test unless the data were not normally distributed; in these instances, the Mann–Whitney *U*-test was utilized for comparison. Comparisons in the matched cohort were done using a paired-sample *t*-test, or Wilcoxon signed rank test, where appropriate.

Kaplan–Meier survival analysis was used to assess the freedom from reoperation and freedom from first catheter reintervention and estimate survival rate. Since some patients experienced multiple events, a modulated renewal approach using the Nelson–Aalen estimator of the cumulative hazard was used to compare catheter reinterventions [9]. End points were time to death, surgical reintervention-conduit replacement and catheter reintervention. Comparisons of survival, reoperation and catheter reintervention rates among the 3 groups were performed by log-rank test. Risk factors for conduit reoperation and catheter reintervention were analysed with univariable and multivariable Cox regression models [reported as hazard ratios (HR), 95% confidence intervals (CI), and P-values). Variables with a *P*-value of <0.1 in the univariable analysis were used for the multivariable stepwise backward Cox regression model, while variables with collinearity were evaluated independently in order to get the best-fitting regression model. A collinearity problem was defined as a variance inflation factor greater than 5 and a tolerance lower than 0.2. Schoenfeld residuals were used to evaluate the proportional hazard assumption of the models; a *P*-value of <0.05 indicated time dependency.

Propensity score-matching method was used to match 2 groups [homografts (*n* = 129) and BJVC (*n* = 45)] on a set of 5 covariates (age, weight, gender, diameter of conduit, orthotopic position—Ross procedure). The propensity score was estimated using a logistic regression model with 1:1 nearest neighbour matching without replacement based on an acceptable calliper width of 0.2 times the standard deviation of the logit of the propensity score [10]. The quality of matching was assessed using the standardized difference in means for the 2 groups pre- and post-matching.

For all tests, a *P* ≤ 0.05 was considered to indicate statistical significance. Statistical analysis was calculated with SPSS software 26.0 (IBM Corporation, Armonk, NY, USA). Propensity score-matching and Nelson–Aalen cumulative hazard analysis were performed with XLSTAT Statistical Software for Excel, version 2022.4.1 (Addinsoft, New York, USA).

## RESULTS

Baseline characteristics in each group are given in Table 1. The operative and postoperative data are given in Table 2.

**Table 1:** Demographics and primary diagnosis of patients undergoing right ventricular outflow tract reconstruction

Variable	Total, *n* = 199	Before matching					After matching			
		Homografts aortic 15, pulmonic 114, *n* = 129	BJVC, *n* = 45	Matrix P Plus N, *n* = 25	*P*-value	SMD before matching	Homografts aortic 8, pulmonic 33, *n* = 41	BJVC, *n* = 41	*P*-value	SMD after matching
Age (years), median (IQR)	7.3 (0.003–28.6)	11 (0.003–28.6)	1 (0.005–14.5)	14.3 (0.005–23)	<0.001	0.98	0.2 (0.003–17.7)	1.2 (0.005–14.5)	0.057	0.03
BSA (m^2^), median (IQR)	0.85 (0.17–2.3)	1.18 (0.17–2.3)	0.39 (0.19–1.4)	1.3 (0.2–2.1)	0.001	0.92	0.25 (0.17–1.9)	0.43 (0.19–1.4)	0.14	0.04
Weight (kg), median (IQR)	22 (2.25–119)	33.7 (2.25–119)	7.8 (2.7–47)	41 (2.8–96)	<0.001	0.90	4.2 (2.25–83)	8.7 (2.7–47)	0.14	0.05
Follow-up (years), median (IQR)	8.4 (0.008–20.5)	11.3 (0.008–20.5)	1.98 (0.18–12.7)	5 (0.008–9)	0.001	0.99	10.7 (0.008–20.4)	1.9 (0.18–12.7)	0.001	1.10
Age at implantation, *n* (%)					0.001				0.19	
<1 year	62 (31.1%)	35 (27.2%)	23 (51%)	5 (20%)			26 (63.4%)	19 (46.5%)		
1–10 years	51 (25.6%)	26 (20.1%)	20 (44.4%)	4 (16%)			12 (29.3%)	20 (48.7%)		
>10 years	86 (43.3%)	68 (52.7%)	2 (4.6%)	16 (64%)			3 (7.3%)	2 (4.8%)		
Sex, *n* (%)					0.34	0.23			0.99	0.001
Male	125 (62.9%)	85 (65.8%)	25 (55.6%)	15 (60%)			25 (61%)	25 (60.9%)		
Female	74 (37.1%)	44 (34.2%)	20 (44.4%)	10 (40%)			16 (39%)	16 (39.1%)		
Diagnosis, *n* (%)					0.44				0.16	
Common arterial trunk	32 (16%)	13 (10%)	16 (35.5%)	3 (12%)			9 (22%)	14 (34%)		
TOF + TOF/APV	61 (30.6%)	51 (39%)	2 (4.4%)	8 (32%)			13 (31.7%)	4 (9.7%)		
DORV	6 (3.1%)	4 (3.1%)	1 (2.2%)	1 (4%)			0 (0%)	1 (2.4%)		
PA	17 (8.6%)	7 (5.4%)	5 (11.1%)	5 (20%)			4 (9.7%)	3 (7.3%)		
Aortic valve disease	47 (23.6%)	39 (30.2%)	6 (13.3%)	2 (8%)			10 (24.4%)	7 (17%)		
ccTGA	17 (8.6%)	7 (5.4%)	7 (15.5%)	3 (12%)			1 (2.4%)	5 (12%)		
Others IAA, HLHC, PS	19 (9.5%)	8 (6.9%)	8 (18%)	3 (12%)			4 (9.8%)	7 (17.6%)		
Conduit diameter (mm), median (IQR)	20 (9–30)	23 (9–30)	14 (12–20)	23 (11–27)	0.001	0.86	14 (9–27)	16 (12–20)	0.51	0.012
Conduit diameter (mm), *n* (%)					0.001				0.16	
<18	79 (39.7%)	85 (65.8%)	29 (64.4%)	7 (28%)			31 (75.6%)	25 (61%)		
≥18	120 (60.3%)	44 (34.2%)	16 (35.6%)	18 (72%)			10 (24.4%)	16 (39%)		
Orthotopic position (Ross), *n* (%)	53 (26.6%)	43 (33.3%)	8 (17.7%)	2 (8%)	0.005	0.30	12 (29.3%)	9 (22%)	0.46	0.016
Anatomic position—Ross, TOF, TOF/APV, *n* (%)	113 (56%)	90 (69.7%)	12 (26.6%)	11 (44%)	<0.001	0.83	24 (58.5%)	13 (31.7%)	0.01	0.53

APV: absent pulmonary valve; BJVC: bovine jugular vein conduit; BSA: body surface area; ccTGA: congenitally corrected transposition of grate arteries; DORV: double-outlet right ventricle; HLHC: hypoplastic left heart complex; IAA: interruption of aortic arch; IQR: interquartile range; PA: pulmonary atresia; PS: pulmonary stenosis; SMD: standardized mean difference; TOF: tetralogy of Fallot.

**Table 2:** Operative and postoperative data

Variable	Total, *n* = 199	Before matching				After matching		
		Homografts aortic 15/pulmonic 114, *n* = 129	BJVC, *n* = 45	Matrix, *n* = 25	*P-*value	Homografts aortic 8, pulmonic 33, *n* = 41	BJVC, *n* = 41	*P-*value
Aristotle score (IQR)	7.5 (7.5–15)	7.5 (7.5–15)	10.3 (7.5–13.8)	7.5 (7.5–12.5)	0.051	10 (7.5–15)	10.3 (7.5–13.8)	0.37
Cardiopulmonary bypass (min), median (IQR)	120 (36–332)	120 (36–275)	128 (51–289)	101 (52–332)	0.050	133 (39–263)	128 (51–289)	0.99
Cross-clamp (min), median (IQR)	60 (0–202)	53 (0–171)	74 (0–202)	0 (0–180)	0.079	74 (0–158)	79 (0–202)	0.82
Hospital LOS (days), median (IQR)	12 (4–147)	10.5 (5–89)	25 (7–147)	13.5 (4–112)	<0.001	17 (5–77)	23 (6–147)	0.072
ICU LOS (days), median (IQR)	2 (1–141)	1 (1–77)	8 (1–141)	2 (1–40)	0.003	8 (1–77)	8 (1–141)	0.59
Ventilation (h), median (IQR)	8 (0–1656)	6 (0–1656)	168 (0–1128)	2 (0–325)	0.01	144 (0–1584)	168 (0–1128)	0.71

BJVC: bovine jugular vein conduit; ICU: intensive care unit; IQR: interquartile range; LOS: length of stay.

### Survival

**Figure 1:**
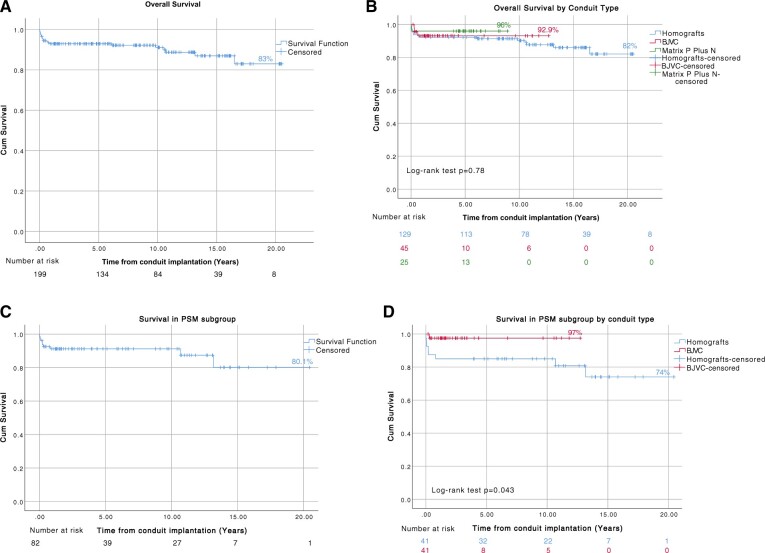
Kaplan–Meier curves showing survival (**A**) all patients, (**B**) by conduit type (log-rank test *P* = 0.78), (**C**) propensity score-matched subgroup and (**D**) by conduit type-propensity score-matched subgroup (log-rank test *P* = 0.043).

The survival rate at 1, 10, 15 and 20 years was 92.9%, 91%, 87% and 83%, respectively (Fig. 1A). Overall, 20 (11.5%) patients died during follow-up (including 30-day mortality), 16 (12.4%) patients in the homograft group, 3 (6.6%) patients in the BVJC group and 1 (4%) in the Matrix P Plus N group (*P* = 0.78). Overall survival according to the type of conduit is shown in Fig. 1B.

Five patients (2.8%) died within the postoperative period of 30 days. The 30-day mortality was 3.1% in the homograft group, 0% in the BJVC group and 4% in the Matrix P Plus N group (*P* = 0.5). No deaths were related to the structural failure of the conduit.

For the propensity score-matched subgroup, the survival rate at 1, 10, 15 and 20 years was 91.2%, 91.2%, 81% and 81%, respectively (Fig. 1C). Overall 9 (11.8%) patients died during follow-up, 8 (20.5%) patients in the homograft group and 1 (2.7%) patient in the BJVC group (*P* = 0.043). Overall survival according to the type of conduit in the PSM subgroup is shown in Fig. 1D.

### Catheter reinterventions

During the study period, 44 conduits (22.1%) underwent at least 1 catheter reintervention, with an incidence of 28 (21.7%), 9 (20%) and 7 (28%) in homograft, BJV and Matrix P Plus N conduits, respectively. The overall median time to first catheter reintervention was 3.4 years (IQR, 7 days to 13 years). Initial catheter reintervention was required in 28 conduits in the homograft group (balloon valvuloplasty, *n* = 21; stent implantation, *n* = 4; PPVI, *n* = 3), 9 in the BJVC group (balloon valvuloplasty, *n* = 9) and 7 in the Matrix P Plus N group (balloon valvuloplasty, *n* = 6; PPVI, *n* = 1). The indications for catheter reintervention were severe conduit stenosis (*n* = 40; 90.9%), with a mean gradient of 62 mmHg, and severe regurgitation (*n* = 4; 9.1%).

**Figure 2:**
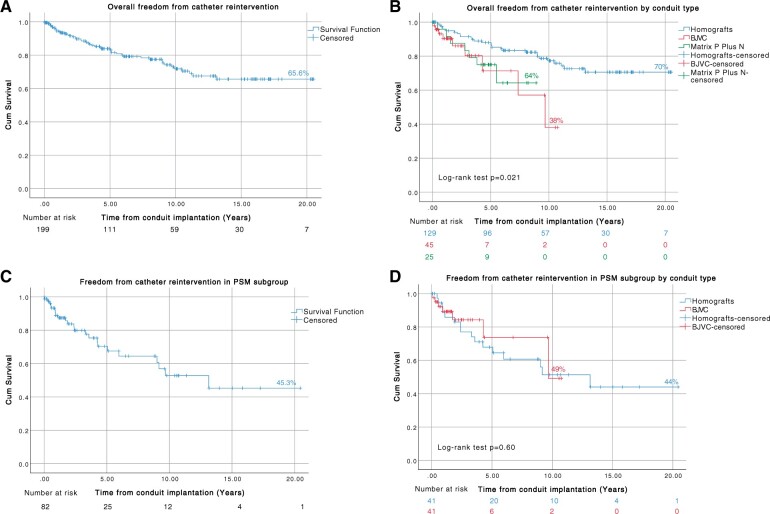
Kaplan–Meier curves showing freedom from first-time catheter reintervention, (**A**) all patients, (**B**) by conduit type (log-rank test *P* = 0.021), (**C**) propensity score-matched subgroup and (**D**) by conduit type-propensity score-matched subgroup (log-rank test *P* = 0.60).

Overall freedom from first catheter reintervention at 5, 10 and 20 years was 83.1%, 71.8% and 65.6%, respectively (Fig. 2A). Freedom from first catheter reintervention at 5, 10 and 20 years was 87.9%, 77.3% and 70% in the homograft group, respectively. Freedom from first catheter reintervention at 5 and 10 years was 71.2% and 38%, in the BJVC group, respectively, and freedom from first catheter reintervention at 5 and 9 years was 75% and 64.3% in the Matrix P Plus N group, respectively. The catheter reintervention rate was significantly different among the 3 groups (*P* = 0.021). Freedom from first catheter reintervention according to the type of conduit is shown in Fig. 2B.

For the propensity score-matched subgroup, 23 conduits (28%) had at least 1 catheter reintervention, with an incidence of 16 (39%), and 7 (17%) in homograft, and BJVC, respectively. The median time to first catheter reintervention was 2.3 years (IQR, 7 days to 13 years). The overall 20-year freedom from first catheter reintervention was 45.3% (Fig. 2C). Freedom from first catheter reintervention at 10 and 20 years was 51.4% and 44% in the homograft group and at 10 years was 49% in the BJVC group. Freedom from first catheter reintervention was similar between homografts and BJVC; no statistically significant difference was found (*P* = 0.60) (Fig. 2D).

### Cumulative (repeated) catheter reintervention

**Figure 3:**
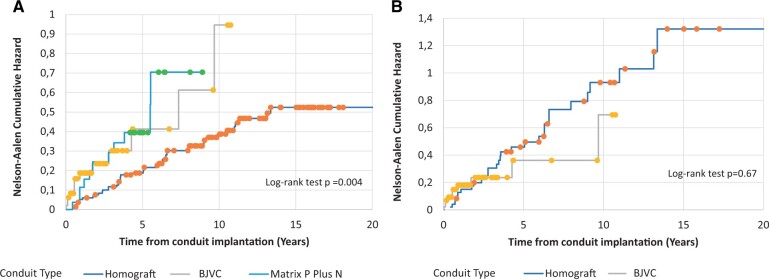
Nelson–Aalen cumulative hazard estimate for freedom from catheter reintervention, (**A**) by conduit type (log-rank test *P* = 0.004) and (**B**) by conduit type-propensity score-matched subgroup (log-rank test *P* = 0.67).

The Nelson–Aalen estimator of the cumulative hazard was used to analyse all repeated catheter reinterventions. A total of 70 catheter reinterventions were performed in 44 conduits. The number of catheter reintervention events before and after matching among homografts, BJV and Matrix P Plus N conduits is summarized in Table 3. The Nelson–Aalen analysis revealed a lower cumulative catheter reintervention hazard for homografts compared with BJV (*P* = 0.006), and with Matrix P Plus N conduits (*P* = 0.01). However, the cumulative hazards were similar between BJVC and Matrix P Plus N conduits (*P* = 0.87) (Fig. 3A).

**Table 3:** Number of catheter reintervention events

Number of reinterventions	Before matching			After matching	
	Homografts (*n* = 129)	BJVC (*n* = 45)	Matrix P Plus N (*n* = 25)	Homografts (*n* = 41)	BJVC (*n* = 41)
1 reintervention	28	9	7	16	7
2 reinterventions	9	3	2	7	2
3 reinterventions	6	1	1	4	1
4 reinterventions	3	0	1	3	0

BJVC: bovine jugular vein conduit.

For the propensity score-matched subgroup, a total of 40 catheter reinterventions were performed in 23 conduits. The Nelson–Aalen cumulative hazards for catheter reinterventions were similar between homografts and BJVC (*P* = 0.67) (Fig. 3B).

### Risk factors

The univariable and multivariable Cox regression analyses identified a smaller conduit diameter <16 mm (HR: 9.1, 95% CI: 4.7–17.60, *P* < 0.001), low body weight < 10 kg (HR: 6.4, 95% CI: 3.4–11.91, *P* < 0.001), younger patient age at conduit implantation <1 year (HR: 9, 95% CI: 4.8–17.47, *P* < 0.001) and the diagnosis of CAT (HR: 2.35, 95% CI: 1–5.5, *P* = 0.004) as risk factors for conduit catheter reintervention. The results of the Cox regression analysis of risk factors for catheter reintervention are presented in Table 4.

**Table 4:** Univariable and multivariable Cox regression analyses to identify risk factors associated with conduit reoperation and catheter reintervention, all patients

Variables	Univariable analysis				Multivariable analysis			
	Reoperation		Catheter reintervention		Reoperation		Catheter reintervention	
	HR (95% CI)	*P*-value	HR (95% CI)	*P*-value	HR (95% CI)	*P*-value	HR (95% CI)	*P*-value
Age at surgery (<1 year)	6.6 (3.03–14.49)	**<0.001**	3.7 (1.85–7.48)	**<0.001**	12.3 (6–25.3)	**<0.001**	9 (4.8–17.47)	**<0.001**
Weight at surgery (<10 kg)	5.3 (2.4–11.6)	**<0.001**	3.68 (1.8–7.39)	**<0.001**	8.2 (4–16.93)	**<0.001**	6.4 (3.4–11.91)	**<0.001**
Conduit diameter (<16 mm)	7.4 (3.2–17.07)	**<0.001**	5.4 (2.64–11.25)	**<0.001**	10.3 (4.8–22.26)	**<0.001**	9.1 (4.7–17.60)	**<0.001**
Anatomic position	1.13 (0.49–2.5)	0.76	0.87 (0.40–1.88)	0.73				
Primary diagnosis								
CAT	2.35 (1–5.5)	**0.04**	2.03 (0.89–4.6)	**0.05**	3.41 (1.6–7.18)	**0.001**	2.75 (1.3–5.49)	** 0.004**
ccTGA	0.58 (0.12–2.6)	0.48	1.1 (0.34–3.5)	0.87				
TOF	1.7 (0.37–7.8)	0.48	0.77 (0.39–1.55)	0.48				
Gender	0.68 (0.31–1.49)	0.34	1.5 (0.76–3.2)	0.21				
Conduit type	1.3 (0.78–2.4)	0.26	0.89 (0.56–1.4)	0.65				
Aortic homograft	2.2 (0.68–7.1)	0.18	2.7 (0.89–8.6)	0.07				

Statistically significant P-values (<0.05) are shown in bold. BJVC: bovine jugular vein conduit; CAT: common arterial trunk; ccTGA: congenitally corrected transposition of grate arteries; CI: confidence interval; HR: hazard ratio; TOF: tetralogy of Fallot.

### Reoperations

**Figure 4:**
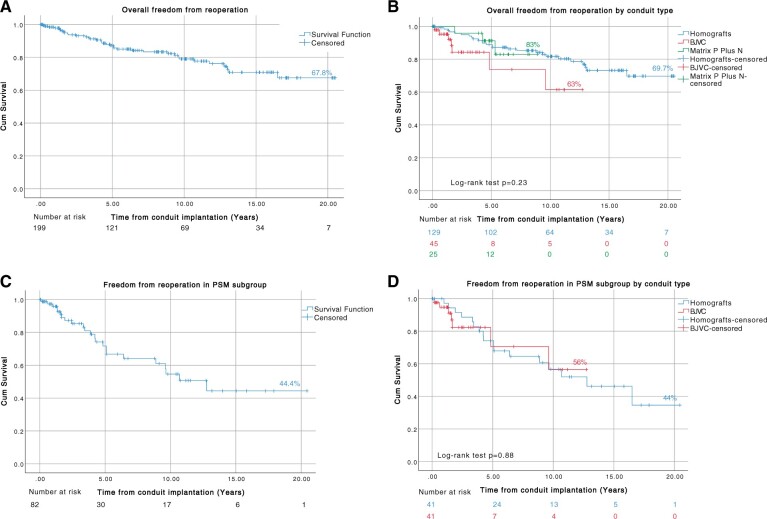
Kaplan–Meier curves showing freedom from reoperation, (**A**) all patients, (**B**) by conduit type (log-rank test *P* = 0.23), (**C**) propensity score-matched subgroup and (**D**) by conduit type-propensity score-matched subgroup (log-rank test *P* = 0.88).

There were 35 (18.1%) conduit reoperations performed. The overall median time to first reoperation was 4.3 years (IQR, 55 days to 16.5 years). Overall freedom from reoperation at 5, 10 and 20 years was 87.6%, 79.4% and 67.8%, respectively (Fig. 4A).

Freedom from reoperation at 5, 10 and 20 years was 88%, 81.7% and 69.7% in the homograft group, respectively. Freedom from reoperation at 5 and 10 years was 75.7% and 63% in the BJVC group, respectively, and freedom from reoperation at 5 and 9 years was 91.3% and 83% in the Matrix P Plus N group, respectively. No statistically significant difference regarding the reoperation rate was observed among the 3 groups (*P* = 0.23). Freedom from reoperation according to the type of conduit is shown in Fig. 4B.

For the propensity score-matched subgroup, 24 (29.2%) conduits underwent reoperation. The median time to first reoperation was 4 years (IQR, 2 months to 16.5 years). The overall 20-year freedom from reoperation was 44.4% (Fig. 4C). Freedom from reoperation at 10 and 20 years was 56.6% and 44% in the homograft group and at 10 years was 56% in the BJVC group. There was no statistically significant difference between the 2 groups (*P* = 0.88) (Fig. 4D).

### Risk factors

The univariable and multivariable Cox regression analyses identified a smaller conduit diameter <16 mm (HR: 10.3, 95% CI: 4.8–22.26, *P* < 0.001), low body weight < 10 kg (HR: 8.2, 95% CI: 4–11.93, *P* < 0.001), younger patient age at conduit implantation <1 year (HR: 12.3, 95% CI: 6–25.3, *P* < 0.001) and the diagnosis of CAT (HR: 3.41, 95% CI: 1.6–7.18, *P* = 0.001) as risk factors for conduit reoperation. Neither the conduit type nor the anatomic position of the conduit had a significant influence on conduit durability. The results of the Cox regression analysis of risk factors for conduit reoperation are presented in Table 4.

## DISCUSSION

For a long time, homografts were considered the gold standard for RVOT reconstruction in patients with congenital heart defects. However, some studies have reported limited availability and poor durability of the homografts [4]. Moreover, the supply of small-sized homografts is also limited [4]. Recently, there has been an increasing number of alternative valved conduits implanted, such as BJVC, Matrix P bioprosthesis, and expanded polytetrafluoroethylene. In fact, there are centres reporting either the superiority [11] or equivalence [12, 13] of BJVC to homografts.

In our study, we compared the durability and performance of 3 different types of conduits (aortic and pulmonary homograft), BJVC and Matrix P Plus N, which were implanted in paediatric, adolescent and young adult patients at our institution as measured by survival, freedom from catheter reintervention, and reoperation. In addition, we performed propensity score-matching analysis between 2 groups of our patients (homograft and BJVC) to look for differences in conduit function. We investigated risk factors for conduit reintervention and reoperation (Table 4).

A survival rate of 91.1% at 10 years and 83% at 20 years in our cohort is satisfactory and comparable to similar reports [14, 15].

Overall freedom from first catheter reintervention in homografts at 10 years was better (77.3%) compared to the BJVC group (38%). However, in the propensity score-matched subgroup, freedom from first catheter reintervention was similar, and no statistically significant difference was found in our study. Additionally, the Nelson–Aalen analysis supported these findings; this reflects the same results in a study from Lewis *et al.* [12].

Overall freedom from reoperation for the entire group at 10, 15 and 20 years was 79.4%, 70.9% and 67.8%, respectively. Freedom from reoperation for propensity score-matched subgroup at 10, 15 and 20 years was 55.7%, 46.2% and 44.4%, respectively. This is comparable to studies from Falchetti *et al.* [14], Tweddell *et al.* [16] and Stark *et al.* [17], who reported 10-year freedom from reoperation between 30% and 82% and 15-year freedom from reoperation between 43% and 54% [12, 17].

In the propensity score-matched subgroup, the freedom from reoperation and freedom from catheter reintervention were equal for homografts and BJVC. Between the 2 groups, there was no statistically significant difference. This compares favourably with similar studies [12, 13], which reported the equivalence of BJV conduits to homografts.

A smaller conduit size <16 mm was associated with the need for conduit reoperation (HR: 10.3, 95% CI: 4.8–22.26, *P* < 0.001) and catheter reintervention (HR: 9.1, 95% CI: 4.7–17.60, *P* < 0.001) in our multivariable Cox regression analysis. This reflects similar results from multiple studies [16, 18, 19] in which small conduits implanted in young, small patients are associated with earlier conduit failure. It is likely that the generally poor function and performance of small conduits are related to somatic growth, resulting in a patient-conduit size mismatch, and may be due in part to accelerated calcium metabolism in children, which can reduce the size of the conduit lumen. In our study, the performance of small conduits (9–16 mm) was similar in the 3 conduit types.

The patients with the diagnosis of CAT were found to be more at risk for earlier conduit reoperation (HR 3.41, 95% CI: 1.6–7.18, *P* = 0.001) and catheter reintervention (HR: 2.35, 95% CI: 1–5.5, *P* = 0.004) in our multivariable Cox regression analysis. Similar results were observed in previous studies [13, 20]. Some studies have found that placement of RV–PA conduit in a heterotopic position increases the risk of conduit failure [3, 13]. This phenomenon may be due to several factors, such as increased turbulence at the proximal part of the conduit, and possible compression of the conduit due to its location behind the sternum. However, our univariable and multivariable Cox analysis failed to demonstrate that heterotopic conduit position was related to earlier conduit reoperation or catheter reintervention, which is consistent with the findings from Lewis *et al.* [12]. In addition, the type of conduit did not affect graft failure leading to conduit reoperation. This finding is similar to data reported by Tweddell *et al.* [16].

The long-term performance of Matrix P Plus N conduits in our study was acceptable. The overall freedom from reoperation and freedom from catheter reintervention in Matrix P Plus N group at 5 and 9 years were 91.3%, 83%, 75%, and 64.3%, respectively. Furthermore, Konertz *et al.* [7] reported overall freedom from reoperation or reintervention of 96 ± 2.5% and 90 ± 4.1% at 3 and 4 years, respectively. Nevertheless, our findings were in contrast with those of Perri *et al.* [21] and Rüffer *et al.* [22], who reported earlier conduit failure in their studies. This difference could be attributed to the surgical technique used in our patients. We implanted the conduit's valve more proximally into the RVOT than is recommended for Contegra implantation. The conduit was tailored to augment the outflow from the right ventriculotomy (also known as a ‘hood’) by using the proximal part of the equine pericardial patch and to establish RV–PA continuity.

### Limitations

The limitations of our study include its single-centre, retrospective analysis of patients with different congenital heart defects operated over a 20-year period and the median follow-up time was much shorter for the BJV and Matrix P Plus N groups compared to homografts.

## CONCLUSIONS

Long-term outcomes of reconstruction of the RVOT using homografts, BJV and Matrix P Plus N conduits were acceptable. The reoperation rate for conduit dysfunction did not differ significantly among the 3 groups (pulmonary, aortic homografts), BJV and Matrix P Plus N conduits. Over time, the need for conduit replacement was higher in smaller conduits and in patients with CAT diagnosis.

## Data Availability

The data sets used and/or analysed during the current study are available from the corresponding author on reasonable request. **Fadi Sabateen:** Data curation; Formal analysis; Investigation; Methodology; Visualization; Writing—original draft; Writing—review & editing. **Vladimír Soják:** Writing—review & editing. **Aref Seif Nagi:** Data curation. **Pavel Valentík:** Data curation. **Michal Šagát:** Data curation. **Matej Nosáľ:** Conceptualization; Methodology; Supervision; Validation; Writing—review & editing. Interdisciplinary CardioVascular and Thoracic Surgery thanks Pranava Sinha, Katarzyna Januszewska, Antonios Kallikourdis, Emre Belli and the other, anonymous reviewer(s) for their contribution to the peer review process of this article.

## ABBREVIATIONS

BJVC: Bovine jugular vein conduit
CAT: Common arterial trunk
CI: Confidence intervals
HR: Hazard ratios
IQR: Interquartile ranges
PPVI: Percutaneous pulmonary valve implantation
RVOT: Right ventricular outflow tract
RV–PA: Right ventricle to pulmonary artery
TOF: Tetralogy of Fallot
